# Numerical Simulation of Electroactive Hydrogels for Cartilage–Tissue Engineering

**DOI:** 10.3390/ma12182913

**Published:** 2019-09-09

**Authors:** Abdul Razzaq Farooqi, Julius Zimmermann, Rainer Bader, Ursula van Rienen

**Affiliations:** 1 Institute of General Electrical Engineering, University of Rostock, 18051 Rostock, Germany (J.Z.) (U.v.R.); 2 Department of Electronics Engineering, The Islamia University of Bahawalpur, 63100 Bahawalpur, Pakistan; 3 Department of Orthopaedics, University Medical Center Rostock, 18057 Rostock, Germany; 4 Department Life, Light & Matter, University of Rostock, 18051 Rostock, Germany

**Keywords:** electrical stimulation, articular cartilage, cartilage–tissue engineering, electrically conductive hydrogels, scaffold, computational modelling

## Abstract

The intrinsic regeneration potential of hyaline cartilage is highly limited due to the absence of blood vessels, lymphatics, and nerves, as well as a low cell turnover within the tissue. Despite various advancements in the field of regenerative medicine, it remains a challenge to remedy articular cartilage defects resulting from trauma, aging, or osteoarthritis. Among various approaches, tissue engineering using tailored electroactive scaffolds has evolved as a promising strategy to repair damaged cartilage tissue. In this approach, hydrogel scaffolds are used as artificial extracellular matrices, and electric stimulation is applied to facilitate proliferation, differentiation, and cell growth at the defect site. In this regard, we present a simulation model of electroactive hydrogels to be used for cartilage–tissue engineering employing open-source finite-element software FEniCS together with a Python interface. The proposed mathematical formulation was first validated with an example from the literature. Then, we computed the effect of electric stimulation on a circular hydrogel sample that served as a model for a cartilage-repair implant.

## 1. Introduction

Articular hyaline cartilage is an inhomogeneous, hierarchically ordered, and multiphasic tissue that covers opposing bone surfaces in diarthroidal joints [1]. It is an avascular tissue comprised of chondrocytes surrounded by the extracellular matrix (ECM) [2]. Chondrocytes are responsible for synthesizing and maintaining the ECM [3], which mainly consists of collagen fibers, proteoglycans, and the interstitial fluid phase [4]. Based on a distinct matrix composition and cellular properties, different zones of articular cartilage can be distinguished [5,6]. The compositional and structural environment of the tissue is altered due to the progression of osteoarthritis or injury [7,8]. Ideally, such alterations should be reversed for the proper functionality of the tissue.

Hydrogels are three-dimensional polymeric networks of high water-uptake capacity, being capable of mimicing native tissue environments [9]. Due to the potential of tailoring the structural and compositional properties of hydrogels toward relevant biological types of tissue, they are considered optimal for becoming a substitute for damaged tissue [10]. In cartilage–tissue engineering, hydrogels should provide adequate mechanical strength, physiological swelling, lubrication, and piezoelectric behavior to replicate the biomimetic environment, similar to native articular cartilage [9,11]. With tissue-engineered hydrogel scaffolds fabricated from biologically derived or synthesized polymeric materials featuring controlled degradation profiles, opening voltage-activated channels can be achieved using external stimuli [12].

Since articular cartilage is essentially a form of a natural polyelectrolyte hydrogel [13,14], electroactive hydrogel scaffolds are a promising approach to mimic its properties to improve cartilage–tissue engineering strategies [15]. The chondrogenic phenotype of chondrocytes inside these hydrogel matrices can be maintained by applying suitable biophysical stimuli [16]. Various biophysical stimuli like mechanical [17], electrical [18,19], and pulsed electromagnetic fields (PEMF) [20,21] were reported for in silico, in vitro, and in vivo applications. In these approaches, electrical stimulation is one of the beneficial methods for enhancing cartilage–tissue repair and regeneration, while it has been less investigated as compared to mechanical stimulation and PEMF [22]. The electrical-stimulation response of articular cartilage has been investigated mainly in vitro and in few in vivo studies. There, the focus was on the medical and biological part of the experiment, while the electrical part has not often been investigated in detail. An option to shed light on the electrical processes are in silico studies. To date, only few studies have been performed in silico [18]. Thus, there is a need for in silico studies to enhance the understanding of the electrical effects and subsequently find an optimal protocol for neocartilage–tissue engineering.

There are two basic methods to apply electrical stimulation to in vitro cultures or in vivo animal models [23]. The first scheme uses electrodes that are in direct contact with the biological sample, while the second scheme consists of an indirect coupling system with electrodes separated from the sample [24]. The input for the case of direct contact can either be a stationary (direct-current) or time-varying (alternating-current) signal, while input for indirect coupling is only the time-varying signal. The indirect coupling can either be inductively coupled (IC) or capacitively coupled (CC) [18,24]. A subtype of the IC is the PEMF, which originates from the natural strain-generated potentials observed in bones [23]. Specifically, it is hypothesized that PEMF causes the bone to deform in a similar manner as due to an external load [25].

There exist subtypes of the basic stimulation methods as well as to optimize the electrical stimulation. For example, in the direct-contact scheme, the electrically exposed sample could be isolated from the reactive products of electrolysis stemming from redox reactions occurring at the electrode surface [26] by using agar salt bridges [27]. In the current study, we suggest a setup that isolates the sample from those reactive electrolysis products. Similarly, for indirect coupling, in order to have optimized stimulation, the capacitive and inductive stimuli can be combined in a single setup [28]. The chondrogenic differentiation of cartilage can be enhanced by the application of either direct or indirect stimulation.

Hydrogels can be used either as acellular scaffolds or as cell-seeded biomaterials for healing the articular-cartilage defect using tissue-engineering approaches [16]. Acellular scaffolds function as cellfree implants replacing weak or damaged cartilage tissue. Efforts are made to engineer acellular biomaterial scaffolds to replicate the architectural features, mechanical properties, and thus biological functions of native cartilage tissue [29,30]. Using cell–biomaterial combinations, the concept is to deliver suitable cell-laden biomaterials as artificial ECM to promote chondrocyte attachment and matrix formation at the defect site [29]. The aim is to present the residing cells’ developmental and microenvironmental cues to trigger chondrogenesis by the presence of the surrounding hydrogel that can be further enhanced by the application of external biophysical stimuli [31,32].

The aim of the current numerical study was to analyze the distribution of ions and the electric potential in a hydrogel scaffold and the surrounding medium that can be used for designing electrical-stimulation experiments and studying the interactions of chondrocyte-seeded hydrogels at the microscale. In Section 2, we briefly discuss the state of the art that has been reported using either direct or indirect electrical stimulation for chondrocytes, cartilage tissue, or cell-seeded hydrogels. In Section 3, an open-source software framework is described for the finite-element simulation of electroactive scaffolds using cartilage–tissue engineering. In Section 4, we report solutions of a validated finite-element model and an extension of the model using circular geometry.

## 2. Electrical-Stimulation Studies of Articular Cartilage

### 2.1. Direct Coupling

#### 2.1.1. In Vivo Studies

As early as 1974, Baker et al. [33] observed the enhancement of the latent potential for the repair of hyaline cartilage in New Zealand white rabbits by using a bimetallic device inserted into articular-cartilage defects. The repair response appeared to stem from proliferating chondrocytes and spread from the defect margin over the entire surface of the defect [33]. Later, they also reported that the regrowing potential of articular hyaline cartilage at the defect site could be enhanced by changing the electrochemical environment by the application of electrical stimulation [34]. Lippiello et al. [35] observed improved repair quality of the articular cartilage by application of a pulsing direct current to rabbit joints in which an osteochondral defect was surgically created.

#### 2.1.2. In Vitro Studies

Electrokinetic transduction in cartilage occurs due to the presence of charged groups of the macromolecules in the ECM, mostly the proteoglycans. Frank et al. [36,37] experimentally and theoretically demonstrated this transduction phenomenon in bovine articular-cartilage tissue. Later, current-generated stress was also measured, and its dependence on the applied current’s amplitude and frequency was characterized [38]. Similarly, Akkin et al. [39] and Youn et al. [40] studied this phenomenon using phase-sensitive optical low coherence reflectometry (PS-OLCR) and differential-phase optical coherence tomography (DP-OCT), respectively.

In the doctoral thesis of Gray, the response of an epiphyseal plate in organ culture was determined over a current-density range of 50 to 1000 μA·cm^−2^ at frequencies between 0.1 and 100 Hz [41]. The motivation for these experiments was to examine the sensitivity of chondrocytes to currents similar in magnitude and frequency to those expected to occur in in vivoloading situations. MacGinitie et al. [42,43] developed an experimental model to observe the effect of electrical stimulation on cartilage tissue manifesting itself in field-induced changes of stress protein and total protein synthesis. Furthermore, the choice of electrical parameters such as frequency and field magnitude that could influence the protein synthesis in cartilage explants were addressed [44]. Nogami et al. [45] investigated the effect of direct current electrical stimulation of 5 μA on mesenchymal cell differentiation into cartilage using bone matrix gelatin and fetal rat muscle.

Since the ability to guide chondrocyte movement may pave the way for strategies to achieve cartilage healing or repair and for further development of cartilage substitutes, Chao et al. [46] reported that cathodal migration occurs for cultured chondrocytes subjected to directly coupled electrical stimulation. Furthermore, an electric field was applied to chondrocytes seeded in a homogeneous agarose culture system and the changes in cell proliferation and ECM biosynthesis were quantified with an emphasis on the cellular signaling pathways responsible for the observed changes [27].

Using the 3D chondrocyte—agarose model system, Akanji et al. [47] investigated the effects of direct current on matrix synthesis and cell proliferation. Kwon et al. [48] investigated the effect of electrical stimulation during chondrogenesis of mesenchymal stem cells (MSCs). Most recently, it was observed by Hiemer et al. [49] that exposure to a directly connected alternating electric field (700 mV, 1 kHz) significantly increased the synthesis of collagen type II in human chondrocytes under hypoxic culture conditions.

### 2.2. Indirect Coupling

#### 2.2.1. In Vivo Studies

Farr et al. [50] used a CC device in 288 patients with knee osteoarthritis and reported that those patients who received electrical stimulation for more than 750 h, profited from pain relief and hence decrease in anti-inflammatory drug use. Moreover, Garland et al. [51] reported that an optimized CC electrical stimulation device for treating knee OA significantly improved symptoms and function without causing any significant side effects.

#### 2.2.2. In Vitro Studies

Rodan et al. [52] demonstrated that an external, capacitively coupled electric stimulation (CCES) applied to chondrocytes in suspension stimulates DNA synthesis. This effect could be attributed to Ca${}^{+2}$ and Na${}^{+}$ fluxes. Fitzsimmons et al. [53] used the CC-pulsed electric field for human chondrocytes and, based on the results, suggested that nitric oxide is involved in the transduction pathway for chondrocyte proliferation, and that its production may be the result of a cascade involving calcium, calmodulin, and cGMP production. The CCES with 60 kHz and 20 mV/cm was found to be a proper and effective inducer of differentiation of human adipose-derived stem cells (ADSCs) into the chondrogenic direction [54]. Later, the same study was extended with a frequency of 1 kHz [55] showing the same principal results.

The group of Brighton et al. conducted many studies on the effect of CCES on chondrocyte proliferation [56,57,58,59,60,61]. In one of their first studies, they observed that articular cartilage chondrocytes from Holstein calf in pellet form showed increased glycosaminoglycan synthesis or increased cell proliferation by appropriate CCES [56]. Similarly, they observed upregulation of gene expression as well as the matrix accumulation of structural cartilage macromolecules (such as type II collagen and aggrecan) with specific CCES in vitro [57]. Later, this study was extended to adult bovine articular cartilage explants, and similar positive results could be reported [58].

Brighton et al. [59] observed significant upregulation of cartilage matrix protein expression and production while simultaneously significantly attenuating the upregulation of metalloproteinase expression by the use of CCES. Thus, it was concluded that the use of electrical stimulation to both diminish matrix destruction and increase matrix production has promising potential to noninvasively treat osteoarthritis patients. The mechanisms through which CCES stimulates matrix production and inhibits matrix destruction were previously unknown. Thus, Xu et al. [60] conducted a study to ascertain that the effect of electrical stimulation does not involve intracellular Ca${}^{+2}$ repositories but solely extracellular Ca${}^{+2}$ influx via voltage-gated calcium channels. At the same time, calmodulin, calcineurin, and the nuclear factor of activated T-cells (NF-AT) decrease rather than phospholipase C and IP${}_{3}$. Finally, they reported the use of reflectance spectrophotometric analysis to demonstrate the structural modification of osteoarthritic articular human cartilage explants [61].

The capability of adipose-derived stem cells was assessed to differentiate into osteocytes, chondrocytes, or adipocytes. Clear patterns of differentiation into three cell lineages were observed after two weeks in differentiating medium [62]. Recently, Vaca-González et al. [63] presented a combined computational and experimental approach to better understand hyaline-cartilage biology and its response to electrical stimulation using different in vitro models in three different scenarios. Initially, cell proliferation and the glycosaminoglycans synthesis of chondrocytes, cultured in a monolayer and stimulated with electric fields, were analyzed. Then, histomorphometric analysis was performed to chondroepiphysis explants that were electrically stimulated [64]. Finally, the effects of electrical stimulation on chondrogenic differentiation of mesenchymal stem cells cultured in hydrogels were assessed [65].

## 3. Electroactive Scaffolds for Cartilage–Tissue Engineering

In general, developing a computational or mathematical model presents a critical prerequisite to design informative and comprehensible experiments [66]. These models can be used to replicate the physical experiments and can be manipulated in ways that would otherwise be too costly and complex. The modeling approach enables to simulate surgical conditions without damaging the biological specimen. Hence, experiments can be easily optimized with respect to electrical-input parameters. Another factor that may make incorporating computational models into experiments seem less daunting is that even solving a single simple equation can improve an experiment design [66], as repeating experiments is difficult due to ethical, logistical, and budgetary constraints. Various cartilage–tissue engineering approaches were experimentally performed but appropriate computational models are still lacking [67,68,69]. Keeping this in mind, we propose an open-source simulation workflow to develop better experiment procedure for cartilage–tissue engineering using electrical stimulation.

**Figure 1:**
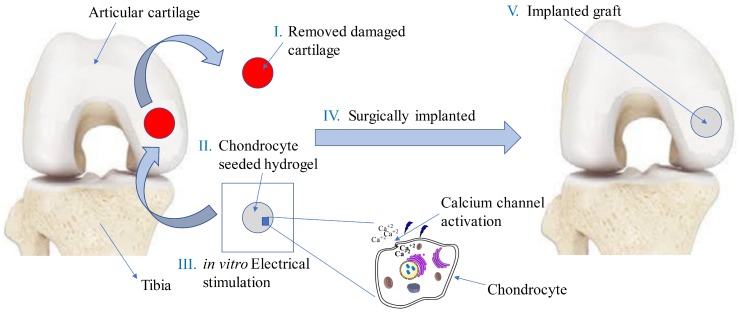
Various steps involved in tissue engineering of knee articular cartilage using electrical stimulation by replacing defect site with chondrocyte-seeded hydrogel (adapted from [11]).

**Figure 2:**
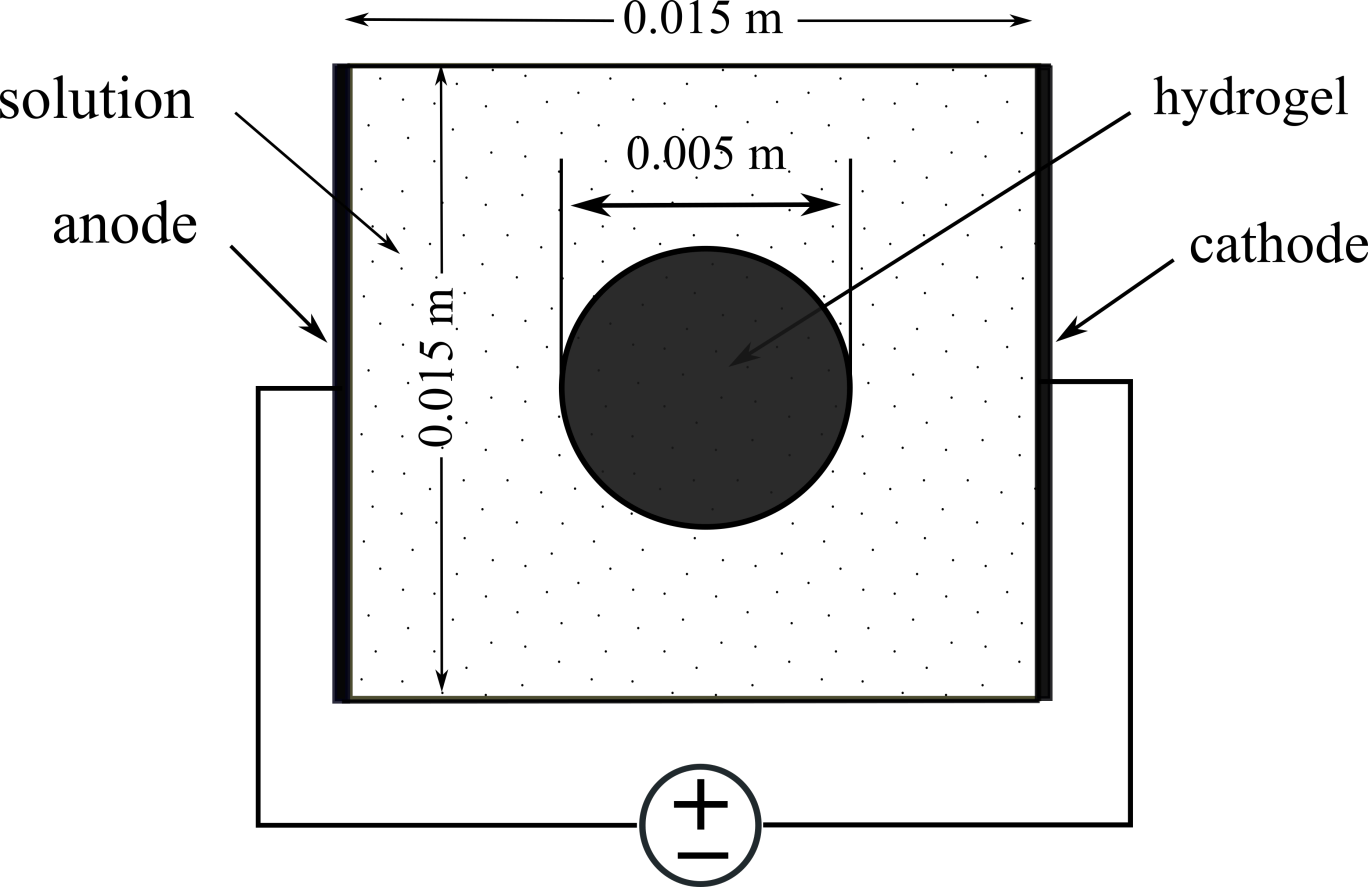
Hydrogel sample immersed in NaCl bath solution under externally applied electric field.

It is known that application of electrical-stimulation results in ion motion and the opening of voltage-gated calcium channels [70]. The increased activity of intracellular-calcium concentration activates the underlying mechanisms that facilitate cell growth, proliferation, and differentiation in a tissue-engineered sample. The complete tissue-engineering approach is schematically described in Figure 1. In our current study, we are only concerned with the in vitro electrical stimulation part of the tissue-engineering approach as illustrated in Figure 2. By means of numerical simulations, these mechanisms can be studied at different-length scales.

The sample consisting of a hydrogel embedded with chondrocytes was immersed in NaCl solution subject to an externally applied electrical stimulation as shown in Figure 2. The implementation of the simulation model consisted of solving nonlinear coupled Poisson–Nernst–Planck (PNP) partial differential equations (PDEs) numerically using FEniCS to find the distribution of the electric potential, and the concentration of anions and cations with the given set of boundary conditions. The distinction between hydrogel and solution phases was realized by using different material parameters for each domain. The most important feature of the hydrogel phase in this model was the presence of bound anionic charges $c^{f}$ with $z^{f}$ valence. The complete mathematical description, the weak formulation, and the parameters for the PNP equations are given in the next sections.

## 4. Materials and Methods

Several mathematical formulations were proposed to simulate the electrochemical behavior of electroactive hydrogels immersed in a solution bath. Based on Flory’s theory [71] and the Donnan equilibrium [72], Shiga et al. [73], and Doi et al. [74] developed models to investigate the hydrogel behavior under an applied electric field. Grimshaw et al. [75] presented a macroscopic continuum theory to explain the dynamic response of hydrogels to electric stimulation. However, these models were not able to precisely simulate the behavior of electroactive hydrogels.

A significant amount of the literature to simulate the electroactive behavior of hydrogels originates from the studies of hydrogel-like biological tissue like articular cartilage. It includes the triphasic theory of Lai et al. [76] and te multiphasic theory of Gu et al. [77], which are based on the classical biphasic theory of Mow et al. [78]. However, these theories are not suitable in simulating electrochemical phenomena in hydrogels, such as ion diffusion and the effect of fixed-charge density on ionic-concentration distribution. Zhou et al. [79] extended the triphasic model to describe the behavior of electroactive hydrogels immersed in the solution, but the computational domain only covered the hydrogel, which limited its applicability.

Based on multiphasic mixture theory, Li et al. [80] proposed a comprehensive model to describe the coupling effects and the multiphasic interactions in the electroactive hydrogels. Similarly, the transport model of polyelectrolyte hydrogels was developed by Wallmersperger et al. [81,82]. Both these models were similar as they consisted of nonlinear coupled PNP equations to describe diffusive ionic species and electric potential, but differed in coupling the electrochemical response to the mechanical equilibrium equation. Since we are only concerned with the electrochemical behavior, either of these two models could be used for result comparison.

In the current study, the model proposed by Wallmersperger et al. [81] was adapted to carry out finite-element simulations. The geometry of the problem is shown in Figure 2, where a square hydrogel sample was first considered for validation instead of the circular hydrogel sample. The model comprised two coupled nonlinear differential equations, i.e., Poisson and Nernst–Planck equations, described below. The PNP equations present several difficulties when computing approximate solutions. It is a strongly coupled system of *n* + 1 nonlinear equations, so computational efficiency plays a critical role in the implementation of the numerical solution, where *n* is the number of mobile ion species.

To date, the PNP model for hydrogels has been simulated using custom programs implemented in individual workgroups [80] that are not generally publicly available. Few of them have also been implemented using commercial software [83]. These models have mostly been implemented for one-dimensional (1D) cases, and a small number of them for the two-dimensional (2D) cases. None of the models has been implemented so far using any open-source software and for the electrical-stimulation response of the hydrogels in context of cartilage–tissue engineering. We have therefore carried out the simulations of electroactive hydrogels for cartilage–tissue engineering using open-source numerical software FEniCS [84].

**Figure 3:**
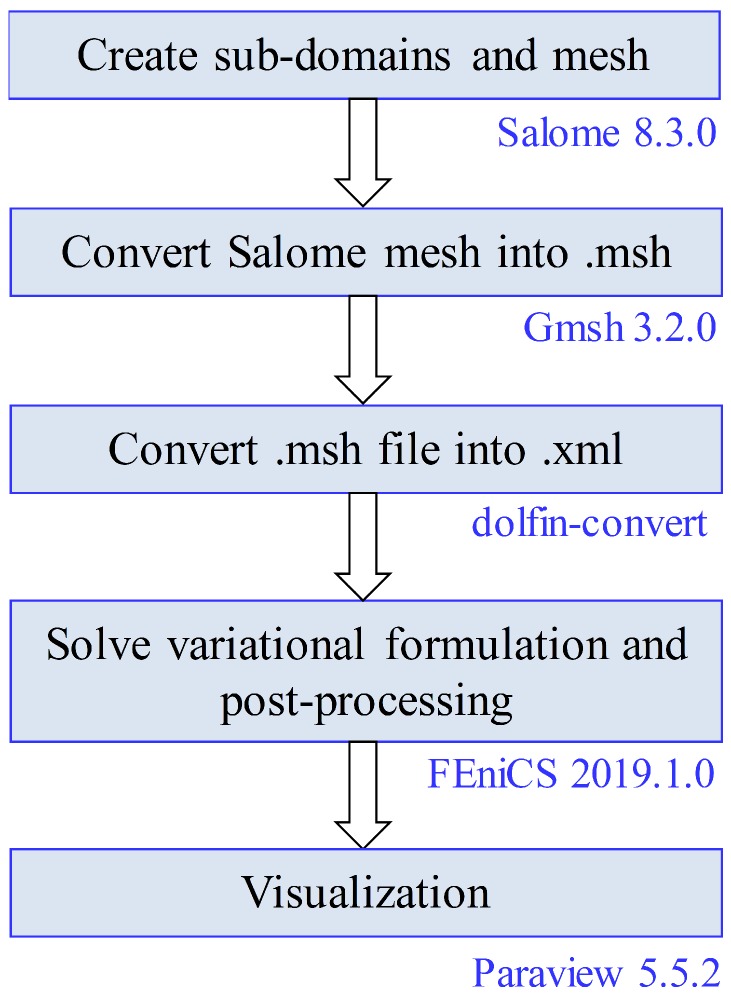
Proposed open-source simulation workflow.

As the geometry considered here is relatively simple, the geometry creation, meshing, and solving the variational problem could all be done using FEniCS, and then visualizing the results using Paraview [85]. For physiologically more complicated geometries, a more comprehensive open-source simulation workflow was implemented as well, which consisted of SALOME (www.salome-platform.org) for geometry creation, Gmsh [86] for meshing, the solution of variational problem using FEniCS, and then result visualization using Paraview. The complete open-source workflow is shown in Figure 3. The results presented here are the same by using either of the two simulation workflows discussed here. The models and the Python scripts used in this study are available in an online repository (https://github.com/arfarooqi/Electro-active-hydrogels).

### 4.1. Poisson Equation

The Poisson equation for the electric potential is derived from Gauss’s law, which states that

(1) ∇·D=ρ

where D and ρ are the electric flux density and the charge density, respectively, which are defined as

(2) $$D=\varepsilon_{r}\varepsilon_{o}E=-\varepsilon_{r}\varepsilon_{o}\nabla \psi$$

(3) $$\nabla \cdot (-\varepsilon_{r}\varepsilon_{o}\nabla \psi )=\rho$$

where $\varepsilon_{r}$ is the relative permittivity of the surrounding medium, $\varepsilon_{o}$ is the vacuum permittivity or dielectric constant, and ψ is the electric potential. As the electric field may be assumed to be curlfree, i.e., the time derivative of the magnetic flux density is negligibly small, we could use a scalar potential as a solution approach [87]. Charge density ρ was related to the mobile and fixed ion concentrations as

(4) $$\rho =F\left(\sum_{k=1}^{n}z^{k}c^{k}+z^{f}c^{f}\right)$$

where *F* is Faraday’s constant, $c^{k}$ represent the ionic concentrations with valence $z^{k}$, and $c^{f}$ is the concentration of fixed ionic species with valence $z^{f}$. From Equations (1) and (2), we get Poisson’s equation as

(5) $$\nabla^{2}\psi +\frac{F}{\varepsilon_{r}\varepsilon_{o}}\left(\sum_{k=1}^{n}z^{k}c^{k}+z^{f}c^{f}\right)=0.$$

Applying homogeneous Neumann boundary conditions at the top and bottom surface of the solution bath, the weak formulation of Poisson’s equation is [88]

(6) $$\int_{\Omega }\nabla \psi \;\cdot \;\nabla v_{\psi }dx=\frac{F}{\varepsilon_{r}\varepsilon_{o}}\int_{\Omega }\left(z^{+}c^{+}+z^{-}c^{-}+z^{f}c^{f}\right)v_{\psi }dx$$

where two ionic species were considered, i.e., cation ‘+’ and anion ‘−’, $v_{\psi }$ is the test function, and dx denotes the differential element for integration over the domain Ω. Here, integration by parts is used and the test function is assumed to vanish on the boundary of the domain Ω.

### 4.2. Nernst–Planck Equation

Total flux $J^{k}$ of ion *k* is composed of three components: diffusion flux $J_{\mathrm{diff}}^{k}$ caused by the chemical potential gradient of ions, electric transference $J_{\mathrm{elect}}^{k}$ caused by the electrical potential gradient, and transfer $J_{\mathrm{conv}}^{k}$ caused by the convection. They are written as [89]

(7) $$\begin{array}{ccc} J_{\mathrm{diff}}^{k} & = & -D^{k}c^{k}\nabla \mu^{k}, \end{array}$$

(8) $$\begin{array}{ccc} J_{\mathrm{elect}}^{k} & = & -z^{k}\mu^{k}c^{k}\nabla \psi , \end{array}$$

(9) $$\begin{array}{ccc} J_{\mathrm{conv}}^{k} & = & c^{k}\nu . \end{array}$$

where $D^{k}$ is the diffusivity of the *k*th ionic species, $\mu^{k}$ is the ionic mobility, and ν is the area-averaged fluid velocity through the hydrogel, relative to the hydrogel’s polymer network. If the effect of the activity coefficient is explicitly taken into account, the diffusion flux becomes

(10) $$\begin{array}{c} J_{\mathrm{diff}}^{k}=-D^{k}\left(\nabla c^{k}+c^{k}\nabla \ln f^{k}\right) \end{array}$$

where $f^{k}$ is the chemical-activity coefficient of the *k*th species. Total flux $J^{k}$ can now be written as

(11) $$J^{k}=-\left(D^{k}\nabla c^{k}+D^{k}c^{k}\nabla \ln f^{k}+z^{k}\mu^{k}c^{k}\nabla \psi \right)+c^{k}\nu .$$

The continuity equation that governs the flux of ionic species throughout the hydrogel and the surrounding solution is given as

(12) $$\frac{\partial c^{k}}{\partial t}+\nabla \cdot J^{k}=0.$$

The first term in Equation (12) represents the change of concentrations with time, while the second term is the combination of the following four contributive terms: the migrative term due to a gradient in the electric potential, the diffusive term resulting from concentration differences, the convective term due to an applied velocity of the solvent, and the source term due to chemical reactions inside the gel-solution domain or at the electrodes.

In Equation (11), it was then assumed that no chemical reactions occurred and convection was neglected. Then, continuity Equation (12) becomes [90,91,92]

(13) $$\frac{\partial c^{k}}{\partial t}=\nabla \cdot \left(D^{k}\nabla c^{k}+z^{k}\mu^{k}c^{k}\nabla \psi \right).$$

Next, mobility $\mu^{k}$ could be determined from the Nernst–Einstein relationship, which relates diffusivity to ionic mobility [93],

(14) $$\mu^{k}=\frac{D^{k}F}{RT}.$$

where *R* is the universal gas constant, and *T* is the absolute temperature. So, Equation (13) becomes

(15) $$\frac{\partial c^{k}}{\partial t}=\nabla \cdot \left(D^{k}\nabla c^{k}+\frac{z^{k}F}{RT}D^{k}c^{k}\nabla \psi \right).$$

Now, the weak formulation of Equation (15) considering two ionic species is obtained by multiplying with a test function $v_{k}$ and integrating over domain Ω,

(16) $$\int_{\Omega }\frac{\partial c^{k}}{\partial t}v_{k}dx=D^{k}\int_{\Omega }\nabla c^{k}\;\cdot \;\nabla v_{k}dx+D^{k}\frac{z^{k}F}{RT}\int_{\Omega }c^{k}\nabla \psi \;\cdot \;\nabla v_{k}dx,\;\left(k=+,-\right).$$

The time derivative can be approximated by using backward (or implicit) Euler difference method [94]

(17) $$\frac{\partial c^{k}}{\partial t}\approx \frac{c_{i+1}^{k}-c_{i}^{k}}{\Delta t}$$

where Δt is the time-step parameter, and $c_{i+1}^{k}$ and $c_{i}^{k}$ represent the concentration of ions for the new and the previous time step, respectively. Using the above notation, Equation (16) was rewritten as

(18) $$\int_{\Omega }c_{i+1}^{k}v_{k}dx-\int_{\Omega }c_{i}^{k}v_{k}dx=\Delta tD^{k}\int_{\Omega }\nabla c^{k}\;\cdot \;\nabla v_{k}dx+\Delta tD^{k}\frac{z^{k}F}{RT}\int_{\Omega }c^{k}\nabla \psi \;\cdot \;\nabla v_{k}dx,\;\left(k=+,-\right).$$

For the numerical solution of the PNP equations, mixed-function space [95] consisting of three scalar functions, i.e., anion, cation, and electric potential was used with Lagrange elements [96,97] of Order 2.

## 5. Results and Discussion

### 5.1. Chemical Stimulation

Initially, the hydrogel was taken out of a solution bath with concentration of 2 mM and put into another with concentration of 1 mM. The concentration of bound anionic groups in the hydrogel was 2 mM and the boundary conditions for the ions at the solution boundary were set to 1 mM. Biological tissue like cartilage and hydrogels exhibited the phenomenon of swelling. It was caused by electric charges fixed to the porous solid. They attracted free ions of the opposite charge, present in the fluid. Fluid flow took place between the hydrogels and the salt solution until equilibrium was reached. The Donnan equilibrium concentration of ions in the hydrogel was calculated using [98,99]

(19) $$\begin{array}{cc} c_{\mathrm{gel}}^{+} & =\frac{1}{2}\left(-c^{f}+\sqrt{{\left[c^{f}\right]}^{2}+4{\left[c_{\mathrm{sol}}^{-}\right]}^{2}}\right)\\ c_{\mathrm{gel}}^{-} & =\frac{1}{2}\left(c^{f}+\sqrt{{\left[c^{f}\right]}^{2}+4{\left[c_{\mathrm{sol}}^{-}\right]}^{2}}\right). \end{array}$$

The corresponding Donnan potential created due to the concentration difference inside and outside the hydrogel could be found either in terms of cation or anion concentrations [100,101,102],

(20) $$\Delta \phi =\frac{RT}{z^{+}F}\ln\left(\frac{c_{\mathrm{gel}}^{+}}{c_{\mathrm{sol}}^{+}}\right)\;\mathrm{or}\;\Delta \phi =\frac{RT}{z^{-}F}\ln\left(\frac{c_{\mathrm{gel}}^{-}}{c_{\mathrm{sol}}^{-}}\right).$$

**Figure 4:**
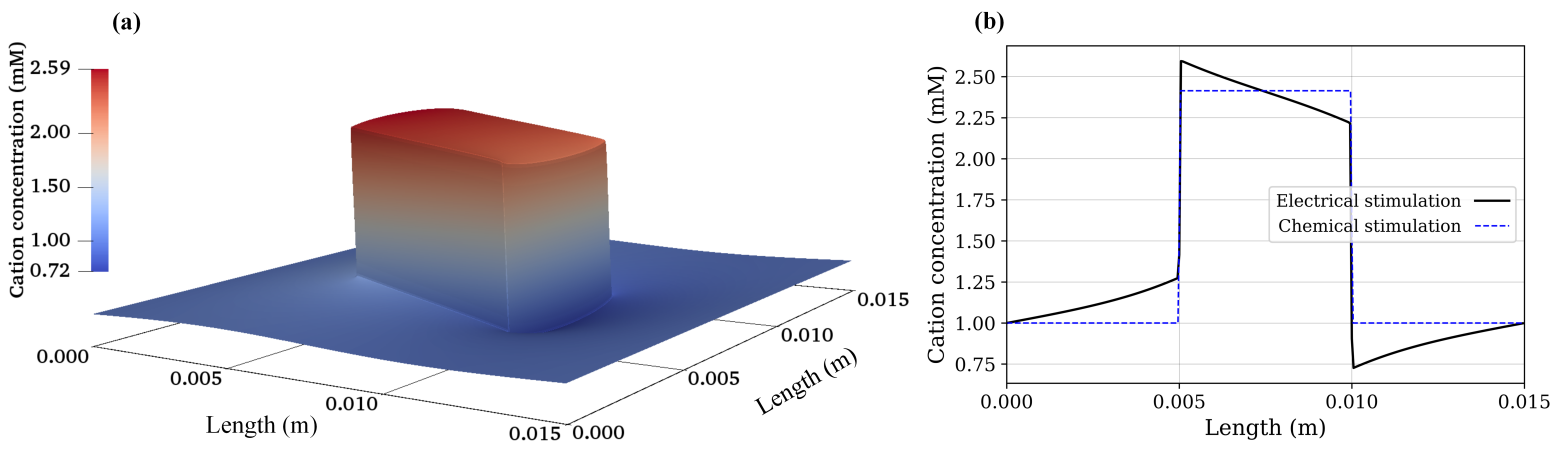
Cation concentration: (**a**) 2D electrical stimulation, (**b**) comparison of chemical and electrical stimulation versus *x*-position at *y* = 0.0075 m.

**Figure 5:**
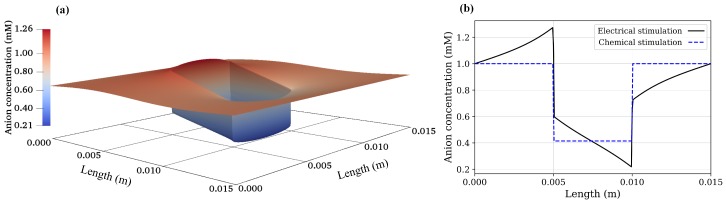
Anion concentration: (**a**) 2D electrical stimulation, (**b**) comparison of chemical and electrical stimulation versus *x*-position at *y* = 0.0075 m.

**Figure 6:**
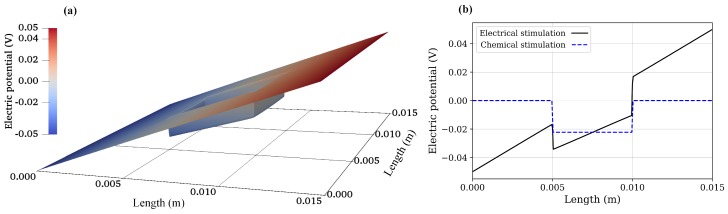
Electric potential: (**a**) 2D electrical stimulation, (**b**) comparison of chemical and electrical stimulation versus *x*-position at *y* = 0.0075 m.

Using Equation (19), the concentration of anions and cations in the hydrogel due to chemical stimulation are 0.4142 and 2.4142 mM, respectively. Similarly, from Equation (20), the value of the Donnan potential was −22.252 mV. Now, the steady-state numerical solution for the chemical stimulation was performed using Equations (6) and (18) considering two ionic species with the concentration of 1 mM for both species at the solution boundaries. For the chemical stimulation, an externally applied electric field was set to zero at the solution boundaries. The parameters used for the numerical simulation are listed in Table 1, and 1D results of the chemical stimulation are shown in Figure 4, Figure 5 and Figure 6, which were the same as by using Donnan theory. These results were also in agreement with the numerical results of Wallmersperger et al. [81].

### 5.2. Electrical Stimulation

For the electrical stimulation, Equations (6) and (18) were again solved numerically for two ionic species using FEniCS with the parameters listed in Table 1. The convective flux was assumed to be zero and no chemical conversion was considered in the equations. The values obtained due to the chemical stimulation were taken as initial conditions for the electrical-stimulation problem, and an external electric potential of 50 mV was applied at the solution boundaries. To achieve the rapid convergence of the solution, local mesh refinement was used, which extended 0.002 m on both sides of the hydrogel–solution interface.

As the electrical stimulation was applied to the bath solution in which the hydrogel was immersed, mobile ions were redistributed in both the hydrogel and the surrounding solution. Due to the fixed-charge groups bound to the cross-linked macromolecular chains of the hydrogel, the diffusion gave rise to the ionic-concentration differences between the interior hydrogel and the exterior bath solution. The graphs for anion, cation, and electric potential distribution are shown in Figure 4, Figure 5 and Figure 6, where 24,668,895 degrees of freedom (DoFs) were used. The 1D graphs for the same quantities extracted from the 2D plots at positions *x* and *y* = 0.0075 m are also shown in the same figures. These results are in agreement to the numerical results of Wallmersperger et al. [81], and in qualitative agreement to the experiment results of Gülch et al. [103].

**Figure 7:**
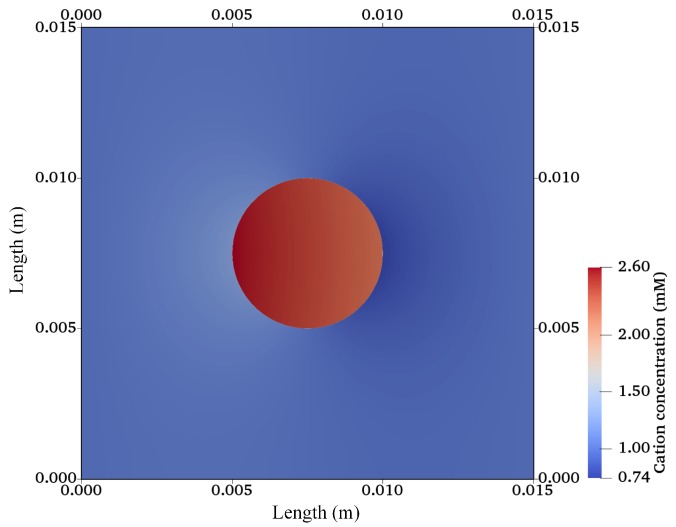
Cation-concentration profile for a hydrogel scaffold immersed in solution.

**Figure 8:**
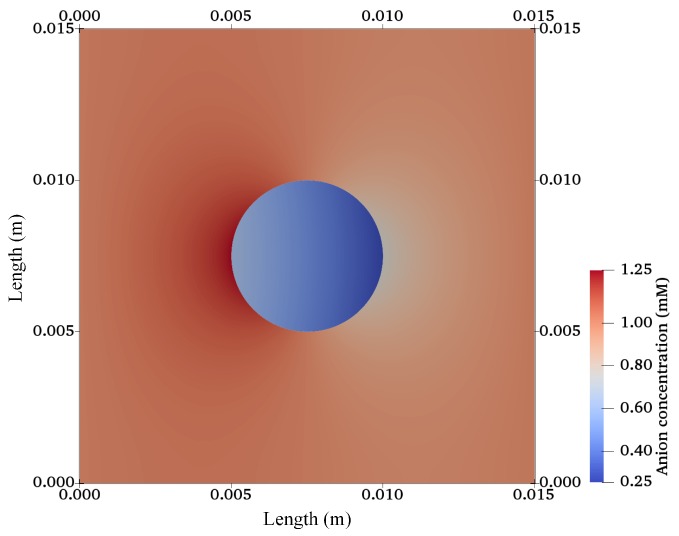
Anion concentration profile for a hydrogel scaffold immersed in solution.

**Figure 9:**
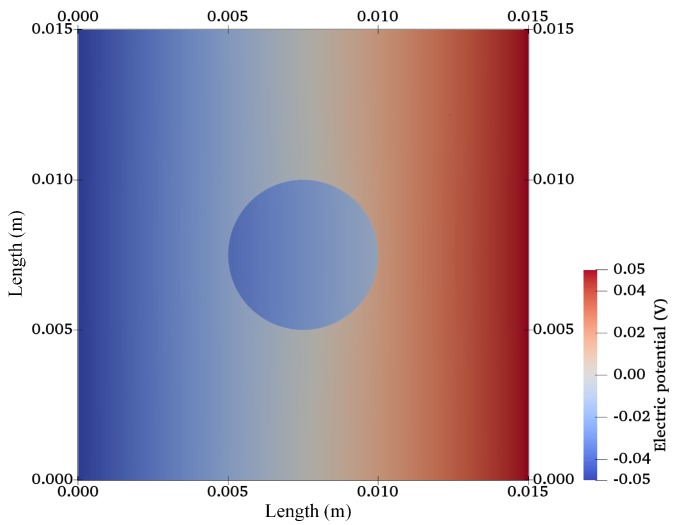
Electric potential distribution for a hydrogel scaffold immersed in solution.

After validation, the finite-element model was extended for a 2D circular specimen (approximation of a 3D cylindrical sample) immersed in solution as used in cartilage–tissue engineering [104] and shown in Figure 1. Figure 7, Figure 8 and Figure 9 show the graphs of the anion, cation, and electric potential distribution in the solution and in the hydrogel scaffold in steady state. Because of the presence of fixed-charge ions in the hydrogel, there was a sharp change in electric potential and in the concentrations of anions and cations to satisfy the electroneutrality condition. In comparison to the initial conditions, the concentrations of cations and anions were slightly higher in the hydrogel on the cathode side as compared to the anode side. Similarly, ion concentrations in the solution near the hydrogel–solution interface increased at the cathode side and decreased at the anode side. From these figures, it can be concluded that the ion concentrations and electric potential in the hydrogel sample could be optimized as the requirement by varying the different quantities. The computation time was ∼58 min with 22,264,695 DoFs on a workstation with 256 GB RAM, Intel(R) Xeon(R) CPU E5-2687W v4 @ 3.00 GHz.

**Figure 10:**
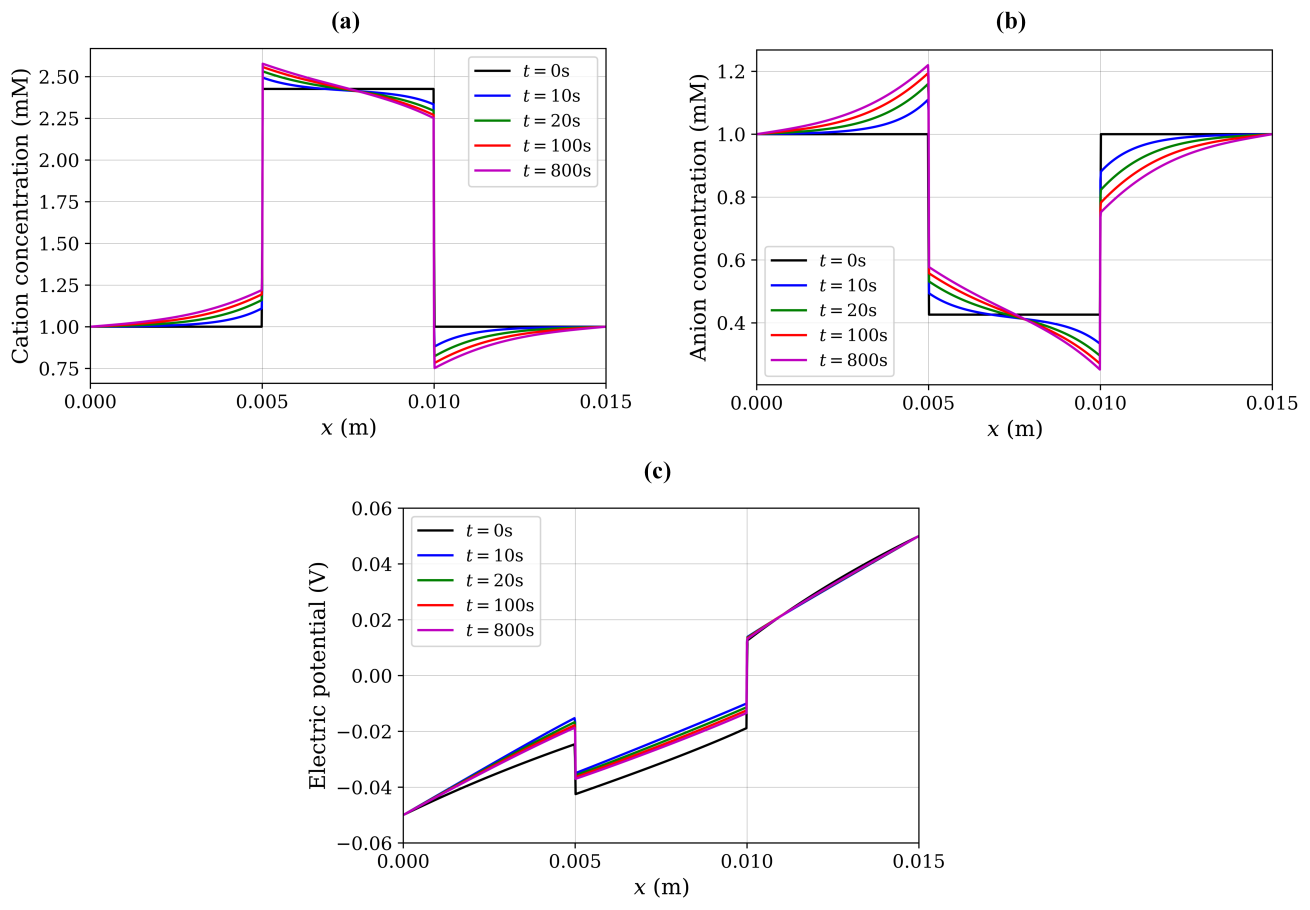
Transient variation of quantities for a hydrogel scaffold immersed in solution: (**a**) cation concentration, (**b**) anion concentration, (**c**) electric potential.

Transient simulations for variations of the ionic concentration and electric potential are also presented in Figure 10. It was observed that, at initial time t=0, the distributions of ionic concentrations in whole computational domain are symmetric, same as from the chemical stimulation without external electric field. As time increased, the diffusive ions redistributed continuously in both the hydrogel and the 1 mM NaCl solution, and the ionic-concentration differences near the hydrogel–solution interfaces became increasingly larger. Ionic diffusion and convection reached the equilibrium state after a specific time, which was dependent on various parameters and conditions, including the electric field, fixed charge density, and NaCl solution concentration. Thus, the concentration of ions and the electric potential in the hydrogel sample could be optimized at various time intervals. For the current case, the steady-state solution for all variables was obtained at around 800 s, after which no further change in the ion concentrations and electric potential occurred. These results follow the same trend as observed by Wallmersperger et al. [81].

Our model still has some limitations that we briefly mention here. The presented numerical method does not explicitly include cells and is thus not capable of estimating an effect on chondrocytes. The numerical method in its current state is also rather expensive and needs tuning on the numerical side. A quantitative comparison to experiment data cannot yet be established since some material parameters like ion mobility and diffusion coefficient have not yet been experimentally measured.

## 6. Conclusions

In this paper, reported research studies to date were summarized using either the direct or indirect electrical stimulation of chondrocytes, cartilage tissue, and cell-seeded hydrogel scaffolds. It is evident that both types of electrical stimulation could be beneficial for cartilage–tissue engineering approaches. However, a complete understanding of the transduction pathways and interaction mechanisms due to electrical stimulation is still lacking for designing an optimized electrical-stimulation protocol.

The development of efficient reproducible computational models is crucial to simulate biological tissue for the better and improved understanding of physiological interactions and therapeutic approaches. On the other hand, modeling the behavior of a hydrogel immersed in a solution bath is challenging due to highly coupled nonlinear PDEs. To date, the behavior of electroactive hydrogels immersed in a solution has been simulated using custom programs implemented in individual laboratories that are not publicly available. A few of the models have also been implemented using commercial softwares, but mostly for 1D cases. None of the models have been implemented so far using any open-source software and for the electrical-stimulation response of the hydrogels in the context of cartilage–tissue engineering. Thus, a 2D open-source computational model has been proposed to study the effect of electrical stimulation on a hydrogel scaffold for cartilage–tissue engineering at the mesoscale. The proposed model was verified against results available in the literature and then extended for a circular geometry by solving the PNP equations using FEniCS. This model can further be extended to study cellular interactions at a microscale. Hence, the current study is a step forward in providing augmented computational models for cartilage–tissue engineering in combination with electrical stimulation.

## Figures and Tables

**Table 1:** Simulation parameters used for simulations [81].

Parameter	Value
Cation valence $z^{+}$	1.0
Anion valence $z^{-}$	−1.0
Bound charge valence $z^{f}$	−1.0
Ion mobility $\mu^{k}$	$3.9607\times 10^{-6}$ m^2^ s^−1^ V^−1^
Ion diffusion coefficient $D^{k}$	$1.0\times 10^{-7}$ m^2^ s^−1^
Faraday constant *F*	9.6487 C mol^−1^
Temperature *T*	293 K
Gas constant *R*	8.3143 J mol^−1^ K^−1^
Vacuum permittivity $\epsilon_{o}$	$8.854\times 10^{-12}$ A s V^−1^ m^−1^
Relatively permittivity $\epsilon_{r}$	100.0
